# Sex and Careers of University Students in Educational Practices as Factors of Individual Differences in Learning Environment and Psychological Factors during COVID-19

**DOI:** 10.3390/ijerph17145036

**Published:** 2020-07-13

**Authors:** María del Carmen Olmos-Gómez

**Affiliations:** Department of Research Methods and Diagnosis in Education, Faculty of Education and Sport Science, University of Granada, 52071 Melilla, Spain; mcolmos@ugr.es

**Keywords:** psychosocial competences, teaching, education, university students, physical activity

## Abstract

The present research was carried out in Spain during the COVID-19 pandemic following emergency school closures in an attempt to avoid the spread of infection. As a result, university students undertaking the final year of education degrees (teaching placements) have been obliged to deliver their teaching from home, adapting their teaching contexts to learning in virtual settings. A novel instrument was designed and validated in order to analyze the impact of learning environments and psychological factors in university students during a period of teaching placements. This took place in an adaptive context (state of confinement). Associations were determined between learning environments and psychological factors in adaptive contexts, in relation to the group to which they belonged (whether undertaking a degree in primary education, physical education and sport, early education or social education), and sex. The present study used a Delphi method, alongside a descriptive and quantitative analysis. The data demonstrate that learning environments differ according to the degree studied. The four analyzed groups revealed significant differences in relation to learning environments and psychological factors in adaptive confinement contexts. The subjects of Primary and Social Education were seen to be related with a greater possibility of being overwhelmed and reporting difficulties. Those more used to physical exercise showed more positive psychological indices. Females reported more negative responses. The conclusion reached is that the results of the present research will enable future additional multi-dimensional analysis to be conducted.

## 1. Introduction

In December 2019, there was an outbreak of pneumonia of unknown etiology in the city of Wuhan, in the Hubei province. A new coronavirus was identified as the causal agent, and it was denominated COVID-19 by the World Health Organization (OMS). Considered to be from the same family as severe acute respiratory syndrome (SARS) and Middle Eastern respiratory syndrome (MERS), COVID-19 is caused by a betacoronavirus called SARS-CoV-2. This affects the lower respiratory tract and manifests as pneumonia in humans [1]. At the time of conducting the present research work, a large section of the global population is in confinement due to the COVID-19 pandemic caused by the SARS-CoV-2 virus [2].

As indicated by Guernsey et al. [3], the phenomenon of online learning has emerged during the COVID-19 health crisis. Many schools have offered virtual learning to their students [4] as a tool for continuing education during the rest of the academic year. These teachers have been previously shown to reject online learning due to the struggles and challenges faced by teachers in trying to balance teaching [5] or difficulties caused by the technology available to students. The type of activities they wish to put into practice may also be a factor as this relates to whether the measures they use and the way in which they structure learning are appropriate [6]. However, given the confinement imposed on all students and the closure of schools, teachers have not had any other option but to adopt this methodology [7]. Without the possibility of in-person teaching, education provision has become more difficult. Nevertheless, educators are now using materials oriented towards virtual learning. Many use systems for learning management and communication infrastructure, such as Google Classroom, Class Dojo, and others, in order to keep in touch through synchronous online classes and learning applications [3,8]. 

In the present day, we live in a world where Internet access is a basic need. Access to a computer is as essential for learning as having paper, pens, and books [3]. In relation to this, the main objective at all times should be to create, develop, and maintain the means for students to construct knowledge in a shared way, through virtual groups and telematic interaction [9]. It is true that teaching-learning processes adapt to their teaching contexts. The growth of networks and development of virtual settings, in combination with videoconferences, has led to the creation of a continuous space within which students and teachers meet and work with ordinary learning resources. Further, these virtual settings strive to favor and promote social interaction by presenting a way in which collaborative learning schemes can be developed between teachers and students. This relationship is mediated by “Computer Supported Cooperative Learning” (CSCL), which favors communication, mediation, and the shared construction of knowledge, as described by Salmerón et al. [9,10,11,12]. Some virtual classroom formats that can be employed for integral education are videos, forums, videocalls, photos, etc. These technologies are characterized by their capacity to save and transmit lessons in an electronic format [13]. The key to success for these adaptive settings will not be in establishing the virtual setting as a setting for learning but in creating tools specifically designed for teachers. These tools will guide and support students as they move towards their desired goals, enabling creativity in both directions [9,14].

On the other hand, the importance of professional development for the student’s use and management of information communication technology (ICT) must be highlighted. According to Galanouli, Murphy, and Gardner [15], the theme can be considered from various perspectives. Such perspectives include the sharing of good practice, investment in infrastructure, equipment, and the development of training programs to elevate skill levels and promote positive attitudes of students towards ICT. Some students may have experienced fear and worry when transferring their classes to online formats. However, the majority have done so quickly and in a short time-frame. Overall, everybody appears to be achieving appropriate adaptive models. The digital gap is more evident than ever [16]. For Rolla [14], the greatest risk is that “you become a talking head that explains things that the students are not following and they give up and just pretend”. According to Inan and Lowther [17], attitudes depend on students’ perceptions regarding the influence and impact of technology on a student’s learning and performance [18]. Thus, slow changes and adaptations within the educational ambit are frequently explained by the tendency of teachers to have conservative attitudes with regard to ICT use and the modification of their own teaching practice. This explains this adaptive change [19].

Lund, Furberg, Bakken, and Engelien [20] suggest the need to abandon a conception of digital competence that is based on the different contexts in which electronic devices are applied. Being a digitally competent teaching professional is not only a matter of being able to adapt to ICT or internalize it but also having the ability to translate technology from one ambit to another, in this way introducing the conception of adaptive contexts [21].

In this way, during placements, teachers of education-related subjects are obliged to impart their teaching from home, adapting their content and teaching skills in an attempt to keep the course running in as normal a way as possible. Further, as previously mentioned, in some cases, education professionals lack knowledge of the way in which to best achieve this. Simon and Nemeth [22] consider that every teacher looking to implement technological tools will require training, support, and resources, in addition to sufficient time to gradually adapt to changes and be able to achieve success [23].

In addition to the emotions arising as a result of being enclosed and the family context, Kyriacou [24] states that teachers tend to experience higher stress levels than workers in other professions. This could affect their wellbeing, motivation, work satisfaction, and commitment, in different ways. According to Collie et al. [25], teachers’ perceptions in relation to work are fundamental for their psychological functioning. The adaptive situation of confinement may cause students to experience psychological effects which generate high levels of stress, anxiety, and depression [26,27].

In this way, students are also dealing with an array of feelings as a consequence of confinement itself, whilst also having to respond to work demands. The intensity and quality of emotional relationships in this professional sector, according to Frenzel et al. [28], will depend on the judgements made by each individual about an event, in regard to its novelty or the capacity of control they have over it.

University students preparing themselves to be schoolteachers live this double dimension of teaching in their teaching placements. Further, psychological settings in adaptive contexts are becoming more interesting to study due to the fact that teachers in training must tackle the context of teaching whilst, at the same time, tackling the challenges of being a student who is still learning [29].

In this sense, work demands are described as physical, psychological, social, or organizational aspects of work, which require sustained effort or physical and/or psychological (cognitive and emotional) skills [30]. Work demands are not necessarily negative, although they can act as sources of stress when extreme effort on the behalf of employees is required to satisfy these demands [31].

As a function of that previously presented, the objectives and novel contribution of the present study are as follows:

(1) Design and validate an instrument in order to analyze the impact of learning environments and psychological factors on university students during a teaching placement in adaptive contexts (confinement). This is important because no instrument currently exists to examine these parameters.

(2) Determine potential associations between learning and psychological settings in adaptive contexts in relation to sex and other grouping variables to which they belong (primary education, physical education and sport, early education, or social education). This is relevant given that studies exist which state that females have a greater risk of suffering depression in confinement contexts [27].

## 2. Materials and Methods

### 2.1. Method

The present study employed a social-analytical and empirical research method. It is descriptive in nature and uses quantitative analysis [32].

### 2.2. Participants

We analyzed group differences between associations in relation to learning environments and psychological factors in adaptive contexts, according to sex (male and female) and the subject being undertaken (primary education, physical education and sport, early education, or social education). Participants were selected through convenience sampling. Fifteen groups were selected (from a group of 15) from groups of education students training to be future teachers. Participants came from the University of Granada in Andalusia, Spain.

In total, 441 students participated in the present study. The final sample was selected through non-probability (convenience) sampling used to select a sample of (28.2%) students enrolled on an Early Learning degree course, (30.8%) students enrolled in a Primary Education course, (19.4%) students enrolled on a Social Education, and (21.6%) students enrolled of physical activity and sport. This provided a total of 441 future teachers from Andalusia (University of Granada, Spain). Students were attending the fourth year of Primary Education, Social Education, Physical Activity and Sport, or Early Learning degree courses and were completing the placement period at the time of conducting the research. Participants had an average age of 22.73 years (Standard deviation (SD) = 3.688). Of these, 34.1% were male and 65.9% were female; 16.24% were females and 10.06% were males undertaking a degree in Primary Education, 10.69% were females and 16.39% males undertaking a degree in Physical Activity and Sport, 20.58% were females and 5. 48% males undertaking a degree in Early Learning, and 18.39% were females and 2.17% males undertaking a degree in Social Education. The questionnaire was administered to individuals who indicated an express desire to participate and provided written informed consent.

### 2.3. Instrument

Each student to participate in the present study was administered the questionnaire, which was based on the questionnaire developed by Olmedo [33,34,35,36]. Development of the tool adhered to general theoretical guidelines for the construction of psychometric tests [37]. Responses were provided along a Likert-type scale which ran from 1 to 4, pertaining to “totally disagree” and “totally agree”. In order to check interpretation and clarity of items, a preliminary version of the questionnaire was administered within a similar educational center to that included in the final study. Modifications were made to the initial design, eliminating elements that were detected as difficult to understand. This led to three of the original 30 items being removed from the questionnaire. The tool addressed basic principles taken from other international models from Italy and the USA. A group of students (n = 41), with similar characteristics to those of the sample used in the present study, were asked to indicate the elements with which they felt they most identified. Following the collection of this information, we proceeded to the process of content validity via an expert panel. Seven experts with PhDs in research fields related to that of the present study evaluated the instrument using the Delphi method [38]. They highlighted any element that they considered to be incomprehensible, whilst also examining the congruence, adequacy and belonging of each item. The final version of the adapted instrument was developed in the exploratory phase. To this end, three rounds of analysis were conducted by the expert panel. The agreement percentage for the first round was 78%, 81% in the middle round, and 91% in the final round.

Following this, exploratory validity analysis was conducted. The final questionnaire comprised 26 questions, divided between four dimensions (factors). The instrument was split into two parts. In the first phase, participants were requested to provide their personal information. This included the reference group to which they belonged, sex, age in years, and religion. Informed consent was provided following receipt of a full explanation about the way in which data collected by the tool would be used. The second part included questions about the frequency with which participants’ opinions were expressed. Opinions were provided in respect to elements expressed on a 4-point Likert scale (from “totally disagree” to “totally agree”). Explanatory factor analysis (EFA) was applied in order to examine the construct validity of the instruments. The Kaiser-Meyer-Olkin (KMO) index was calculated and produced a highly acceptable result of 0.907. The sphericity test was used to ensure that an acceptable significance level was achieved, with a value of 0.000 being obtained. This permitted us to proceed to factor analysis. We applied the Scree criterion and yielded 4 factors. Finally, from the variance table for the varimax rotated matrix component, we were able to obtain variance results for all analyzed variables. Results of the previously described analysis identified the existence of four factors, explaining 75.673% of overall variance.

We estimated reliability through the Cronbach alpha. This produced a value of 0.934 from the scores provided on the scale when using testing for ordinal data. Considering the data according to factors, for factor 1 a value of 0.92 was obtained, with values of 0.918, 0.913, and 0.895 obtained for factors two, three, and four, respectively [39].

**Figure 1:**
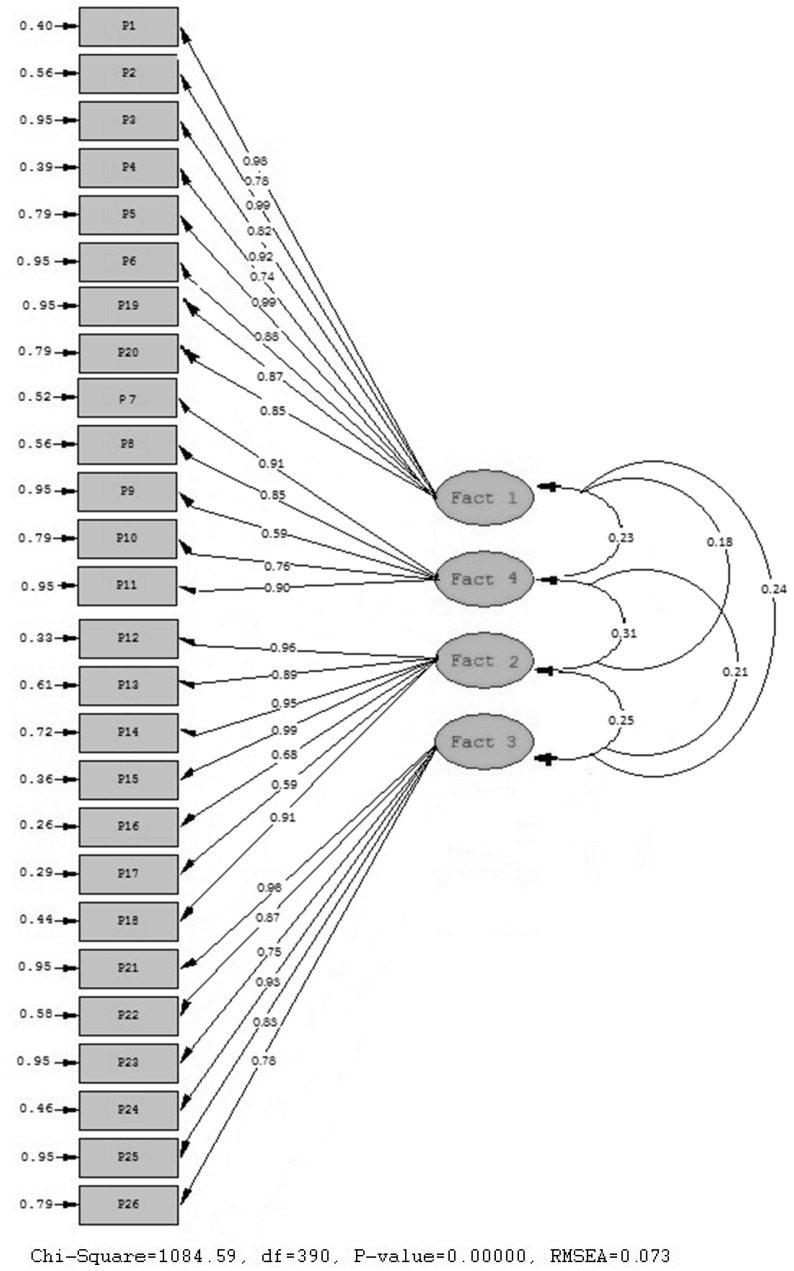
Schematic (path graph) of Questionnaire of Psychological and Learning Environments in adaptive Contexts (QPLEC).

Next, we conducted confirmatory factory analysis (CFA) using the method of structural equation modeling (SEM). From this, we examined the multivariate regression coefficients produced from the structural equations [40]. Evaluation of data fit to the developed model was conducted in accordance with the following criteria: Chi-squared and degrees of freedom (χ^2^/df), comparative fit index (CFI) and root mean square error of approximation (RMSEA). CFA results confirm adequate fit of the data to the model (Figure 1). This model is based on both exploratory factors and a relevant theoretical model. Parsimonious fit was χ^2^/df = 1084.59 / 390; *p* < 0.001. A model was formed with 90 degrees of freedom (df) and with a maximum probability. The resultant *p*-value was lower than 0.05, whilst the CFI (comparative index of goodness of fit) was 0.90, TLI (Tucker-Lewis index) was 0.91, and RMSEA [41] was 0.073 (90% CI = 0.053–0.080). Data were analyzed using the statistical software package SPSS 20 (International Business Machines Corporation (IBM), Chicago, IL, USA, 2011), LISREL v9.1 (Scientific Software International, Princeton, NJ, USA, 2010), and PANTH GRAHF (Scientific Software International. Princeton, NJ, USA, 2010) [42]. The literature suggests that model fit can be considered as adequate when χ^2^/df < 5, CFI > 0.90 and RMSEA < 0.08 [43]. Our values, therefore, comply with requirements established in the literature for establishing acceptable fit. As can be observed in Figure 1, all regression weights were greater than 0.05, whilst the covariance between factors varied between 0.29 and 1.00. The evaluation carried out according to SEM methodology confirms that the coefficients produced support the theory employed for configuring the measurement model [44]. Coefficients produced from the multivariate regression analysis were examined through examination of the covariance matrix of observed variables, each grouped component was expressed as a latent variable, and the various elements associated with each factor were expressed as observable variables. The LISREL 9.1 program was used to complete this analysis. Observed and latent variables were structured as shown in Figure 1. Confirmatory analysis served to confirm the theoretical factors which significantly influence observed variables in the way predicted based on EFA. This explains 4 obtained factors: Factor 1: Personal management of learning; Factor 2: Essential values reported by university students; Factor 3: Basic psychological factors, and Factor 4: Learning environments in virtual contexts.

The majority of existing questionnaires that have been developed to examine learning environments or psychological factors have not been administered in a situation as novel as the current situation of confinement in which we find ourselves. Likewise, they ignore the variable pertaining to values included in the present questionnaire. In addition, the present study is novel in that it is directed towards university students undertaking the final phase of their course, engaging in a period of practical development to be trained as teachers at the time of their participation in the study [45].

We developed an instrument which established four dimensions (factors) for analyzing the learning environments and psychological factors of implicated groups, whilst also considering the interactional influence of sex. The first dimension is called personal learning management and is related with perceptions of motivation, reflection, and planning. This dimension is linked with a factor previously presented by Olmedo [33] and Martínez [34]. The second dimension is associated with the essential values developed in response to confinement. It is based on a questionnaire developed by Gervilla [36], focusing on aspects related to the categories of body, affect, decision making, uniqueness and opening. These values are seen as being respectful of collaboration and cooperation between students. The third dimension describes basic psychological factors in relation to the state of confinement. These factors are to be considered in relation to the adaptive context in which university students are currently having to develop their practice [35,45]. They are mainly centered on factors linked to sleep, emotions, and physical disorders, such as headaches, exhaustion, or anxiety. Finally, the fourth dimension refers to the use of telematic learning environments for the learning of future teachers [33,46]. Here, we consider the use of digital media at a personal and professional level, in terms of frequency of use, and knowledge about and learning of such media.

### 2.4. Procedure

#### 2.4.1. Data Collection Procedure

Questionnaires were administered online during April 2020. The department of social responsibility at the University of Granada approved the questionnaire. Prior to its completion, the following statement was provided to participants: “Before beginning to respond to the following questionnaire it is crucial that you provide us with informed, unequivocal and express consent regarding data privacy and protection regulations, stating that you accept that those responsible for the study will handle your personal data in compliance with that set forth in the (EU)2016/679 regulation, of the 27th of April (GDPR)”. Questionnaires were distributed and administered through the Google Forms platform. Participants were not fully informed about the ultimate purpose of the study in order to avoid the effects of social desirability. However, the following indication was also provided: “The following questions aim to study university students undertaking teaching placements, some aspects relate to the confinement we are all currently suffering due to COVID-19”.

#### 2.4.2. Data Analysis

Once internal consistency and factorial validity of the questionnaire was established, in accordance with the process previously discussed, we proceeded to carry out the most appropriate statistical procedure. For this, the Levene test was conducted and homogeneity of variance was examined, providing confirmation that parametric testing was appropriate [47]. In order to study the relationships between variables as proposed in the second of our objectives, a multi-variate analysis was conducted for multiple comparisons. Analysis of variance (ANOVA) with multiple factor levels was used to evaluate the difference between participating groups and differences according to sex.

## 3. Results

Table 1, Table A1 and Table A2 (Appendix A) demonstrate the extent to which participants are dealing with the context of confinement. It can be seen that groups comprising students undertaking degrees in early education, as well as physical activity and sport, are adapting better, as shown through better values for psychological factors. They also have more positive perceptions of their learning environment than groups comprising primary education and social education students. It is also notable that females undertaking social education degrees reported more negative aspects at a psychological level. In contrast, males belonging to the degree of physical activity and sport students reported greater learning in their context.

Table 1 shows ANOVA results in relation to grouping variables and sex, as well as the interaction between them. The effect size was analyzed using the eta-square tests (eta-square values greater than 0.14 are considered to show a large effect) [48,49]. Initially, multivariate analysis of variance (MANOVA) testing indicated significant differences and large effect sizes for sex, significant differences and large effect sizes regarding degrees, and significant differences for the interaction between sex and degrees and large effect sizes.

Multivariate tests enable simultaneous analysis of the relationship between different levels of the same variable and the relationship *p* between the levels of two different variables [50]. These tests identify covariance effects, enabling us to statistically study the influence of independent groups (with four levels: PE = Primary Education, EL = Early Learning, SE = Social Education, and PES = Physical Education and Sport) and sex (with two levels: male and female) using mean scores at an individual level. Results indicate significant differences and large effect sizes in relation to sex, degrees, and the interaction between sex and the degrees variable. The sample size and proportion of explained variance (in the ANOVA) [51] (see Table A2), with regard to the interaction variable of sex and group, produced a result of more than (η^2^ > 0.14). This suggests that this proportion of differences can be attributed to the effect of learning environments and psychological factors. Typically, eta-square values greater than 14 are considered to show a large effect, but the literature explain that eta-square tests (low effect r = 10; medium effect r = 0.30; high effect r = 0.50) [48,49,52]. The high value could be due to both the gender representation within the sample and the concrete influence of degree choice, given that different levels and various measures derived from different populations were studied [52,53,54,55]. Similarly, significant differences are shown with regard to sex as a function of study factors and sample size (ANOVA) [56]. With respect to the sex variable, significant differences were found (η^2^ > 0.14), indicating that observed differences can be attributed to effects of the studied variables. This outcome should be kept in mind. When we consider the squared sample size between different groups of degree courses, (η^2^ > 0.14) is due to the squared sample.

The fit of ANOVA results to the data reveals significant associations in relation to the learning environments and psychological factors, determined according to sex and degrees. Analysis also focused on contexts during COVID-19, examining sex in relation to differences degrees in education practices variables. Significant results were produced in relation to: “When I am faced with a problem I evaluate its importance, analyse its causes, and dedicate time and effort to solve it”; “I am incapable of solving problems by myself, I draw on tools from my PLE [personal learning environment] which aid me in this communication (*p* = 0.001)”; “I am able to specify what I want to achieve and where I want to get to as a function of my goals (*p* = 0.002)”; “I have developed better links with my family and friends during confinement” (*p* = 0.003); “during confinement I have frequently suffered from headaches” (*p* = 0.020); and, “during confinement I have felt bored, sad, wanted to cry, etc.” (*p* = 0.020). Thus, it can be seen that learning environments and psychological factors in adaptive contexts, when considered in relation to the groups to which they belong (whether they are undertaking a primary education, physical education and sport, early education, or social education course) and sex, produced large effects sizes in all cases.

## 4. Discussion

The present research, the psychometric instrument was designed using the Delphi method. Content validity of the scale was analyzed. A panel of 7 experts used existing literature [38] in order to establish variable names. It is worthwhile to mention that prior studies have focused on analyzing psychological factors or learning-related factors, yet few studies exist which encapsulate both factors and are adapted to the current state of confinement. Thus, it is hugely important to be able to offer a reliable and empirically tested instrument in order to continue advancing. We find ourselves in a situation from which we would all like to learn how to improve in all senses and find a way in which to be better prepared.

Additional analysis conducted on the questionnaire included exploratory factor analysis (EFA) and instrument reliability. These analyses were also carried out using the previously presented statistical programs. All elements were found to be reliable. Next, confirmatory factor analysis (CFA) was performed to confirm the model through examination of acceptable indices (CFI and TLI) [40,43]. Additionally, analysis of content validity demonstrated consistency with the scale used in relation to the majority of proportions produced by the developed instrument. A novel characteristic of the elaboration and validation of this instrument is the inclusion of elements, specific to the confinement context, centered on learning environments and psychological factors, in addition to student values [33,34,35,36]. These aspects are included in the consideration of a necessary tool for the evaluation and improvement of university education for future teachers.

In order to carry out the present study, we analyzed the inter-relation between sex and factors which affect learning environments in adaptive contexts in accordance with different groups (university students undertaking a Primary Education degree, university students undertaking an Early Education degree, university students undertaking a Social Education degree, and university students undertaking a Physical Activity and Sports Sciences degree). All participants were in the last year of their course, thus undertaking teaching placements. ANOVA testing demonstrated significant differences and large effect sizes in relation to the frequency of participant responses, with respect to learning environments and psychological factors as a function of group and sex.

A total of 75.25% of the participants reported that, when faced with a problem, they are able to resolve it through tools available in their PLE (chats, email, Facebook, Tuenti, etc.). Current means of communication enable new learning systems to be established via existing channels, permitting young people to develop their social, educational, and personal interaction. In recent years, Western society has experienced significant changes due to the generalized use of information and communication technology, as well as its increased use amongst adolescents [57]. In total, 83.5% consider themselves able to specify goals, establishing where they want to get to. Further, 78.75% considers that they have developed stronger ties with family and friends during confinement. This corroborates the idea extolled in current literature about the family [58,59,60], educational setting [61,62,63], and place of work [64,65,66], with these being considered essential for individual’s learning development. It is highlighted that 71.87% of individuals suffered from frequent headaches during confinement, whilst 69.02% have felt bored, sad, and the need to cry, etc. These symptoms mirror those detected in other studies of the immediate impact of the COVID-19 pandemic on mental health or quality of life [67,68,69]. Psychological factors have been most strongly established in relation to social education students, followed by those undertaking early education and primary education studies. Those in sports courses were seen to be the least affected. This may indicate that these students are protected from boredom and sadness by being in permanent contact with physical activity and sport. This has been established in various research studies that suggest that belonging to a sporting environment [70,71,72] helps individuals in their critical development, including Castro et al.’s conclusions on their study in which they highlight that a task-involving climate and engagement in physical activity are both associated with lower levels of life stress and higher levels of academic performance [73]. Likewise, prior research [74,75] has demonstrated the importance of psychological needs to the development and wellbeing of individuals. This has strong implications for educational science, motivation, and social development adapted to specific context [76].

Analysis through the ANOVA test demonstrated significant differences and varied effect sizes in relation to participants’ response frequency regarding variables pertaining to learning environment and psychological factors, as a function of sex and the educational course being undertaken. Thus, differences were found with regard to sex when examining the variables, 1, 2, 4, 6, 10, 11, 12, 14, 15, 17, 18, 19, 24, and 25. Females were found to exhibit more problematic psychological factors, especially those undertaking a social education degree. Within the consideration of degree specialty, all variables except for 10, 12, and 13 emerged, with males embarking on physical education and sports courses exhibiting better development of their personal learning environment (PLE). Such development predicts better future success. On the other hand, the group pertaining to those undertaking social education exhibited suffering greater psychological problems during the development of their professional practice in confinement.

The data demonstrate that learning environments different according to the degree subject being studied. Students undertaking the degrees of Primary and Social education report greater difficulties and being overwhelmed more often than others. In the case of Social Education, this course is directed towards disadvantaged contexts, with students focusing on parents in difficult economic situations who, as stated by Goedhart et al. [77], are largely influenced by parenthood, poverty, low literacy levels, poor education, and little experience of the digital society, regardless of migration history. This explains the fact that students undertaking this degree also report greater difficulty when acting in this new age of learning. The same was also seen, unfortunately, with primary education students. It may be relevant that a large proportion of children are still found in parts of the country who do not find it easy to access the internet. Some families may share a computer, while others may not have a computer at all. Associations, the government, companies, and educational systems must be engaged to find ways of providing all students with computers and tablets. In this way, we will facilitate the learning of all young people, despite the precarious situation of some families [3]. It is not new that digitalized society today demands that specialized social services continually adapt to the changing needs of users and transformations in society itself. In the future, responding to these needs will help parents to work with their children in the technological setting and in settings, such as the present one, in other words, pandemics, health crises, etc. This is important as these are all settings in which students find themselves to be disadvantaged [77].

The present research has also uncovered other aspects. For instance, university students have had to incorporate themselves into a complicated adaptive system. In this sense, they have responded with autonomy and have adjusted well, though effects can be seen at a number of levels. In this sense, work demands are described as physical, psychological, social, or organizational aspects of work which require sustained effort or physical and/or psychological (cognitive and emotional) skills [30]. Work demands are not necessarily negative, but they can provide a source of stress when excessive effort is required by employees to meet them [31]. Females are found to be more affected by headaches, sadness, boredom, wanting to cry, etc. This confirms our second objective by identifying groups at risk of suffering depression during confinement [27].

It is necessary to highlight some limitations of the present study. Firstly, the sample should be extended to include all centers for university education, not only in Spain, but also throughout Europe. We also emphasize the need to conduct more studies capable of providing evidence about the effect of factors in contexts that are adaptive but do not involve confinement. It will be useful to identify the individuals and social principles required for this concept to come to life. Another of the limitations experienced by the present study was due to difficulties in accessing the sample as a result of the situation described throughout the study. This complicated participant involvement as it could not be conducted in-person; however, it is important to note that data collection was still completed within an acceptable time-frame.

## 5. Conclusions

From the present study, we conclude that we have developed a good (internal consistency and factorial validity) tool, thus fulfilling the first study objective. In this sense, we have expanded upon results obtained regarding the advantages of the designed tool. Through provision of this instrument, the present research also favors learning environments and positive psychological factors. This can be appreciated in the four analyzed groups as significant differences were reflected between the various items associated with the learning environment and psychological factors in adaptive confinement contexts.

Hyun-Joo et al. [78] state that depressive symptoms in teachers not only influence the quality of their teaching practice but also their work-related wellbeing, including their professional motivation and stress. Likewise, Kyriacou [79] established that stress in teachers can be defined as the experience of unpleasant and negative emotions, resulting from some aspects of their work [31]. Previously conducted research studies conducted by Ecker et al. [80] demonstrate that social ambiguity and anxiety conditions arise during periods of crisis or pandemics. These may include malaise and sadness. According to Altena et al. [81], sleep pressure and homeostatic drive are key for sleeping well, thus avoiding headaches and sadness. Boredom can be tackled by engaging in physical activity during the day (but not late at night), with this also improving sleep quality [82]. This conclusion is reiterated in the present group of Physical Activity and Sport Science students who, being more accustomed to exercise, most effectively dealt with the psychological issues that arose throughout the study.

During the COVID-19 pandemic, following news updates for more than 3 h a day has been associated with higher levels of anxiety, with this having negative repercussions in comparison to those who less exposed themselves to news updates in relation to COVID-19 [83].

A study conducted by Yu and Yu [84] reports that the majority of research studies on students’ participation and the success of electronic learning, in which the impact of students’ attitudes has been observed, ultimately focused on the commitment of students to using the system. When a student’s understanding of learning is incorporated as a part of system design, it is more likely that the resultant system will adapt to individual needs.

In conclusion, the pandemic provides an opportunity for young people to develop and perfect their resilience and adaptability, enabling them to appreciate the value of social responsibility and personal sacrifice in order to protect the most vulnerable. More research is needed into online replacements for face-to-face lessons. It is also necessary to provide training to teachers so that they can prepare for change in extreme situations, such as that which we are currently living [85]. It is crucial that we validate young people’s experiences during this global crisis and listen to their creative solutions, in order to tackle this new reality and connect with each other. We must up-skill so that students can use these new skills to create a connected society that is characterized by solidarity; in this way, we will emerge equipped in this changed world [86].

The conclusion reached herer is that the results of the present study will permit future multi-dimensional analyses to be conducted [87].

## Figures and Tables

**Table 1:** ANOVA and effect size (η^2^) sums of aggregated scales for Questionnaire of Psychological and Learning Environments in adaptive Contexts (QPLEC) by sex and field of study.

Factors		M	SD	CI (95%)		F	*p*	*η* ^2^
				Lower Limit	Higher Limit			
Personal management of learning	Careers	3.04	0.798	2.89	3.14	0.392 3.251 1.196	<0.005 <0.005 >0.005	>0.14 <0.14 >0.14
	Sex	2.98	0.696	2.61	3.21			
	Careers × sex	3.02	0.854	2.72	3.18			
Learning environments in virtual contexts	Careers	3.01	0.998	2.98	3.08	0.679 3.132 1.150	>0.005 <0.005 >0.005	<0.14 >0.14 >0.14
	Sex	2.89	0.746	2.74	3.02			
	Careers × sex	2.91	0.846	2.62	3.25			
Basic psychological factors	Careers	2.72	1.023	2.31	3.20	0.978 7.123 2.459	<0.005 <0.005 <0.005	>0.14 >0.14 >0.14
	Sex	2.81	0.997	2.51	3.07			
	Careers × sex	2.77	1.006	2.39	3.22			
Essential values reported by university students	Careers	3.15	0.984	3.09	3.30	0.967 6.165 2.189	<0.005 >0.005 <0.005	>0.14 >0.14 >0.14
	Sex	2.89	0.886	2.74	3.2			
	Careers × sex	3.04	0.976	2.83	3.23			

The critical alpha level was adjusted for multiple testing to reduce the type I error (α). Thus, the α-value was divided by the number of pair comparisons for each ANOVA.

## Appendix A

**Table A1:** Means and standard deviations for QPLEC by careers and sex.

Items		Careers	Male		Female	
			Mean	SD	Mean	SD
Factor 1: Personal management of learning	V.1.	PE	3.30	0.483	3.33	0.486
		EL	2.83	0.718	2.12	0.825
		SE	3.13	0.354	3.30	0.364
		PES	3.38	1.188	3.13	0.354
		Total	2.92	0.784	3.15	0.464
	V.2.	PE	3.20	0.480	3.10	0.473
		EL	2.75	0.754	3.32	0.500
		SE	3.13	0.991	3.00	0.500
		PES	3.63	0.916	3.13	0.991
		Total	2.95	0.804	3.15	0.675
	V.3.	PE	3.10	0.568	3.08	0.566
		EL	3.17	0.835	3.11	0.601
		SE	2.88	0.835	3.33	0.502
		PES	2.50	1.195	2.88	0.835
		Total	3.25	0.868	3.12	0.653
	V.4.	PE	3.20	0.632	3.12	0.602
		EL	3.08	0.996	3.22	0.667
		SE	3.00	1.069	3.33	0.707
		PES	3.38	0.916	3.00	1.069
		Total	2.95	0.928	3.19	0.801
	V.5.	PE	3.00	0.667	3.15	0.670
		EL	2.92	1.084	3.02	0.707
		SE	3.00	0.756	3.11	1.000
		PES	3.38	1.302	3.09	0.756
		Total	2.84	0.973	3.12	0.800
	V.6.	PE	3.10	0.316	3.14	0.318
		EL	2.92	0.793	3.11	0.333
		SE	3.13	0.354	3.22	0.441
		PES	3.25	0.886	3.13	0.354
		Total	2.87	0.704	3.15	0.368
	V.7.	PE	2.90	0.568	2.80	0.565
		EL	2.83	1.193	2.89	0.601
		SE	2.62	0.916	3.03	1.118
		PES	3.25	1.035	2.62	0.916
		Total	2.68	0.962	2.85	0.881
	V.8.	PE	3.15	0.816	3.25	0.826
		EL	3.00	1.128	3.18	0.866
		SE	2.62	0.518	3.23	0.707
		PES	3.63	0.916	2.62	0.518
		Total	2.84	0.886	3.10	0.748
Factor 4: Learning environments in virtual contexts	V.9.	PE	2.90	0.738	2.96	0.408
		EL	2.75	1.055	2.89	0.782
		SE	2.62	0.518	2.89	0.928
		PES	2.93	1.069	2.62	0.518
		Total	2.71	0.867	2.81	0.749
	V.10.	PE	3.30	0.483	2.90	0.538
		EL	2.92	0.793	3.32	0.502
		SE	2.75	0.707	3.04	0.510
		PES	3.38	0.916	2.75	0.707
		Total	2.87	0.777	3.04	0.599
	V.11.	PE	3.10	0.568	3.23	0.668
		EL	2.83	0.835	3.11	0.601
		SE	3.25	0.463	3.02	0.501
		PES	3.50	1.069	3.25	0.463
		Total	2.92	0.784	3.12	0.516
Factor 2: Essential values reported by university students	V.12.	PE	2.90	0.738	2.90	0.738
		EL	3.17	0.835	3.14	0.823
		SE	3.63	0.518	3.11	0.333
		PES	3.50	1.309	3.44	0.527
		Total	3.05	0.928	3.63	0.518
	V.13.	PE	3.00	0.667	3.06	0.765
		EL	3.08	0.996	3.11	0.601
		SE	3.50	0.535	3.33	0.707
		PES	3.00	1.309	3.50	0.535
		Total	3.13	0.906	3.31	0.618
	V.14.	PE	3.30	0.483	3.14	0.495
		EL	3.17	1.115	3.33	0.500
		SE	3.63	0.518	3.33	1.000
		PES	2.75	1.165	3.63	0.518
		Total	3.21	0.905	3.42	0.703
	V.15.	PE	3.10	0.738	2.98	0.6.08
		EL	3.08	0.996	3.11	0.782
		SE	3.25	0.463	3.44	0.726
		PES	2.75	1.165	3.25	0.463
		Total	3.05	0.868	3.27	0.667
	V.16.	PE	3.00	0.667	3.06	0.767
		EL	3.33	0.888	3.00	0.707
		SE	3.63	0.518	3.44	0.527
		PES	3.00	1.309	3.63	0.518
		Total	3.24	0.883	3.35	0.629
	V.17.	PE	3.50	0.527	3.44	0.499
		EL	2.92	0.900	3.56	0.527
		SE	3.13	0.641	3.22	0.667
		PES	2.38	0.916	3.13	0.641
		Total	3.00	0.838	3.31	0.618
	V.18.	PE	3.20	0.789	3.10	0.541
		EL	2.42	0.996	3.22	0.833
		SE	3.00	0.535	2.67	0.866
		PES	2.38	1.188	3.00	0.535
		Total	2.74	0.950	2.96	0.774
Factor 1: Personal management of learning Factor 3: Basic psychological factors	V.19.	PE	2.90	0.876	2.96	0.915
		EL	2.83	1.030	2.89	0.928
		SE	3.25	0.707	3.11	0.601
		PES	2.38	1.188	3.25	0.707
		Total	2.84	0.973	3.08	0.744
	V.20.	PE	2.70	0.949	2.68	0.885
		EL	2.42	0.996	2.67	1.000
		SE	2.88	0.641	2.56	1.014
		PES	2.00	1.309	2.88	0.641
		Total	2.50	1.007	2.69	0.884
Factor 3: Basic psychological factors	V.21.	PE	3.40	0.699	3.44	0.558
		EL	2.58	1.165	2.78	0.726
		SE	3.25	0.707	3.10	0.972
		PES	2.13	1.246	3.25	0.707
		Total	2.84	1.079	3.15	0.834
	V.22.	PE	3.50	0.707	3.56	0.726
		EL	2.83	1.193	3.00	1.118
		SE	3.00	0.926	2.54	0.735
		PES	2.38	1.188	3.02	0.926
		Total	2.95	1.064	3.19	0.939
	V.23.	PE	2.90	1.101	2.89	1.167
		EL	2.42	0.900	2.78	0.667
		SE	2.63	1.061	2.23	0.996
		PES	1.88	1.126	2.63	1.061
		Total	2.47	1.059	2.77	0.951
	V.24.	PE	3.40	0.699	3.44	0.726
		EL	2.50	1.087	2.78	0.972
		SE	2.87	0.835	2.44	0.756
		PES	2.05	1.195	2.02	0.835
		Total	2.71	1.063	3.04	0.871
	V.25.	PE	3.40	0.966	3.44	1.014
		EL	2.33	0.985	2.67	0.866
		SE	2.75	1.035	3.75	1.055
		PES	2.03	1.069	2.10	0.999
		Total	2.63	1.101	2.89	1.054
	V.26.	PE	2.70	1.160	2.44	0.726
		EL	2.42	0.900	2.25	0.886
		SE	2.25	0.886	2.54	0.905
		PES	1.88	1.126	2.44	0.726
		Total	2.34	1.021	2.67	1.118

QPLEC according to sex and careers (PE = Primary Education, EL = Early Learning, SE = Social Education, PES = Physical Education and Sport).

**Table A2:** ANOVA and effect size (*η*^2^) for questionnaire QPLEC by sex and Field of study (Careers).

Factors	Items	Sex			Field of Study (Careers)			Sex × Careers		
		F	*p*	*η* ^2^	F	*p*	*η* ^2^	F	*p*	*η* ^2^
Factor 1: Personal management of learning	1. I use tutoring tools in order to teach more effectively.	8.432	0.006	0.199	0.580	0.017	0.451	3.006	0.044	0.210
	2. When I receive a lot of information, I am able to examine it and isolate what most interest me, ordering it as a function of my goals.	5.960	0.020	0.149	0.007	0.000	0.936	2.190	0.067	0.162
	3. I reflect before selecting the most adequate digital tools (apps, chats, YouTube, forums, social networks, blogs, wikis, etc.) for my learning and professional development.	3.143	0.085	0.085	0.572	0.017	0.455	1.239	0.099	0.311
	4. I plan my teaching work as follows: I prioritize, I think about the time it will take me, the resources I will need, and the results I hope to achieve.	6.301	0.017	0.156	0.382	0.011	0.541	2.229	0.053	0.164
	5. I am able to manage my leisure time, and, for this, I use tools from my personal learning environment (PLE) which help me (such as planners, etc.).	2.143	0.052	0.059	0.209	0.006	0.650	0.867	0.071	0.468
	6. When I am faced with a problem I evaluate its importance, analyse its causes, and dedicate time and effort to solve it; so I used my PLE to solved it.	9.670	0.000	0.367	0.033	0.001	0.856	6.597	0.001	0.368
	19. I am a person who is hopeful of improving, and positive thinking has helped me in confinement.	5.153	0.030	0.132	0.344	0.010	0.561	1.929	0.053	0.145
	20. My spirituality has grown in confinement.	3.051	0.090	0.082	0.010	0.000	0.921	1.192	0.095	0.327
Factor 4: Learning environments in virtual contexts	7. I use tools from my PLE (such as chats, forums, social networks, blogs, wikis…) to relate with others for academic purposes (such as diffuse information, share acquired knowledge, present work…).	2.331	0.036	0.064	0.597	0.017	0.445	0.976	0.079	0.415
	8. I use tools from my PLE (such as chats, forums, social networks, blogs, wikis, etc.) to relate with others for social purposes (diffuse information, share acquired knowledge, present work, etc.).	2.652	0.053	0.072	0.093	0.003	0.762	1.181	0.094	0.332
	9. I share information for professional purposes, using tools from my PLE to achieve this (such as Apps, chats, YouTube, forums, social networks, blogs, wikis, etc.).	0.988	0.057	0.028	0.295	0.009	0.590	0.457	0.039	0.714
	10. What I see, hear or read online (chats, digital libraries, etc.) has been of use to me in my teaching.	4.479	0.042	0.116	3.356	0.090	0.076	2.678	0.062	0.191
	11. My PLEs (digital tools) improve my work and help me in my activities as a student, teaching and in my daily life.	5.404	0.026	0.137	0.021	0.001	0.885	1.854	0.056	0.141
Factor 2: Essential values reported by university students	12. I am able to specify what I want to achieve and where I want to get to as a function of my goals.	15.003	0.000	0.306	3.724	0.099	0.062	6.244	0.002	0.355
	13. I know how to define future short- and long-term goals and organize myself to reach them, even in exceptional situations.	3.397	0.074	0.091	2.767	0.075	0.105	2.120	0.061	0.158
	14. When I have a problem, I try to resolve it in the most satisfactory way possible and try to learn from the experience.	4.819	0.035	0.124	0.196	0.006	0.661	1.758	0.074	0.134
	15. New projects motivate me, without the need for anybody to intervene, and I consider that I am able to carry them out.	5.569	0.024	0.141	0.572	0.017	0.455	2.073	0.052	0.155
	16. I have taken care of my diet, hygiene, and cleanliness during confinement, which has helped me to feel better.	1.277	0.066	0.036	1.188	0.034	0.283	1.154	0.092	0.341
	17. I have developed better links with my family and friends during confinement.	15.230	0.000	0.309	0.853	0.024	0.362	5.600	0.003	0.331
	18. I have enjoyed diverse forms of cultural activities (art, music and other) during confinement.	4.898	0.034	0.126	0.023	0.001	0.879	1.772	0.071	0.135
Factor 3: Basic psychological factors	21. During confinement, I have regularly felt apathy, tiredness, or fatigue.	7.842	0.008	0.187	0.352	0.010	0.557	2.731	0.059	0.194
	22. During confinement, I have had difficulties sleeping and recurring thoughts.	4.733	0.037	0.122	1.127	0.032	0.296	2.048	0.062	0.153
	23. During confinement, I have felt anxiety or depression.	7.198	0.011	0.175	0.239	0.007	0.628	2.503	0.076	0.181
	24. During confinement, I have frequently suffered from headaches.	9.575	0.004	0.220	1.231	0.035	0.275	3.740	0.020	0.248
	25. During confinement, I have felt bored, sad, wanted to cry, etc.	9.037	0.005	0.210	1.082	0.031	0.306	3.736	0.020	0.248
	26. During confinement, I have suffered hyperactivity, obsession, nervousness, etc.	3.211	0.082	0.086	0.880	0.025	0.355	1.665	0.093	0.128

The critical alpha level was adjusted for multiple testing to reduce the type I error (α). Thus, the α-value was divided by the number of pair comparisons for each ANOVA.
